# Integrating Millimeter Wave Radar with a Monocular Vision Sensor for On-Road Obstacle Detection Applications

**DOI:** 10.3390/s110908992

**Published:** 2011-09-21

**Authors:** Tao Wang, Nanning Zheng, Jingmin Xin, Zheng Ma

**Affiliations:** Institute of Artificial Intelligence and Robotics, Xi’an Jiaotong University, Xi’an 710049, China; E-Mails: nnzheng@mail.xjtu.edu.cn (N.Z.); jxin@mail.xjtu.edu.cn (J.X.); mazheng1985@gmail.com (Z.M.)

**Keywords:** visual attention, monocular vision, millimeter wave radar, autonomous ground vehicle, obstacle detection

## Abstract

This paper presents a systematic scheme for fusing millimeter wave (MMW) radar and a monocular vision sensor for on-road obstacle detection. As a whole, a three-level fusion strategy based on visual attention mechanism and driver’s visual consciousness is provided for MMW radar and monocular vision fusion so as to obtain better comprehensive performance. Then an experimental method for radar-vision point alignment for easy operation with no reflection intensity of radar and special tool requirements is put forward. Furthermore, a region searching approach for potential target detection is derived in order to decrease the image processing time. An adaptive thresholding algorithm based on a new understanding of shadows in the image is adopted for obstacle detection, and edge detection is used to assist in determining the boundary of obstacles. The proposed fusion approach is verified through real experimental examples of on-road vehicle/pedestrian detection. In the end, the experimental results show that the proposed method is simple and feasible.

## 1. Introduction

For the engineering development of autonomous mobile robots such as autonomous ground vehicles (AGVs), and unmanned aerial vehicles (UAVs), real-time and reliable obstacle detection in their surroundings is a premise in order to execute precision navigation and control. Due to their complex and dynamic working environments, mobile robots are necessarily equipped with different types of sensors to deal very well with the issues of environmental perception and recognition. Multi-sensor fusion can take advantage of different sensing superiorities of sensors like infrared, vision, sonar, radar, and laser range finder to acquire perceptive information about their surroundings as exactly and completely as possible, and it has been already applied in the realms of industry and the military. In recent years, the development of AGVs has been attracting more and more attention in the World due to its very interesting prospects. Because the on-road driving circumstances are very complex and constantly changing, obtaining timely reliable detection of on-road obstacles is difficult but crucially important for safe navigation and control of the AGV. Considering the all-weather working capability and powerful detection capability of MMW radar and the low cost of a monocular vision sensor, this paper attempts to construct a novel radar-vision fusion architecture for navigating mobile vehicles.

Many scientists have researched applications of MMW radar in the field of precision navigation, especially military applications such as missiles mounted with radar-infrared integrated seekers or satellites equipped with radar detectors for space observation. Recently with its decreasing cost and smaller size, MMW radar as an automotive radar is playing more and more important role in the development of active safety and autonomous navigation systems. To our knowledge, Grimes *et al.* [1–4] were the first to carry out an extensive investigation of automotive radar and discuss in detail its configurations and different potential applications for vehicles, which provided a guidance of later relevant development, and made great efforts on the development of applying radar systems to automobiles. Chuck *et al.* [5] integrated radar with a velocity sensor to use Kalman filters for localization and navigation of vehicles. Clark [6] employed a mechanical scanning MMW radar to extract the surrounding features and combined it with a GPS for AGV navigation. Even though MMW radar has great advantages such as detecting moving objects fast and providing the long-range detection and exact velocity measurement, MMW radar cannot recognize the shapes and sizes of the detected targets. On the other hand, vision systems can easily obtain the contour of the targets within the short-distance sensing region of a visual sensor. Farid *et al.* [7] adopted a vision system based on optical flow for autonomous 3D localization and control of small UAVs. Budi *et al.* [8] utilized block matching technology for real-time tracking and identification of moving pedestrians through a single camera in an outdoor environment. Many researchers have studied vision-based navigation [9,10]. It seems that only a vision sensor could solve perfectly all the problems of environment perception for autonomous navigation application. Nonetheless, because of the state-of-art of computer technology and the drawbacks of the vision sensor like being easily influenced by illumination levels, reliable and real-time vision-based navigation under a complicated and dynamic environment is hard to implement. Therefore, because of the complementary advantages of MMW radar and monocular vision sensor, radar-vision fusion has been receiving more and more attention in these years. Seong *et al.* [11] took a MASRAU0025 24 GHz MMW radar to do data association and moving object tracking by nearest clustering of original radar data. Alessandretti *et al.* [12] fused two 24 GHz scanning radars and vision sensors to improve the detection of vehicles and guard-rails. Wu *et al.* [13] employed stereo-vision and a 24 GHz radar for information fusion and utilized extended Kalman filter (EKF) for vehicle contour tracking and obstacle avoidance. Fang *et al.* [14] exploited the depth information obtained from radar for the segments of the target in the image, and effectively reduced computational loads. Bauson [15] from Delphi Electronics and Safety presented a concept of integrated radar-vision sensor for the active safety system of vehicles. Shigeki *et al.* [16,17] utilized the reflection intensity from MMW radar to do radar-vision spatial calibration and did research on moving obstacle segmentation using MMW radar and image sequences. Bertozzi *et al.* [18] integrated a camera, and a radar with an inertial sensor to perform road obstacle detection and classification. Although there are already many researches on fusion radar and vision [11–18], this paper provides an approach for the fusion of radar and a monocular camera for on-road obstacle detection from a systematic point of view so as to obtain better comprehensive performance, which will decrease the demands of each individual part.

In recent years, commercial MMW radars have become more available and affordable thanks to their lower cost. They generally directly give out the measurement information of the detected target, which has already clustered the original radar data together as the point targets for users’ convenience. In this paper, a commercial bi-mode MMW radar (Delphi ESR 76–77 GHz) and a single Basler camera were used to detect real obstacles and determine their boundaries. The Delphi ESR directly provides the information of detected point targets, which is different from that used in the research mentioned above [11,16,17] where generally original data from the radar is used. The main contributions of this paper are described as follows: firstly, through analysis of drivers’ attention mechanisms and visual consciousness, a three-stage fusion scheme simulating the drivers’ visual information processing mechanism is proposed in order to progressively improve the detection, speed up obstacle detection and reduce false alarm rates for autonomous navigation. Second, with no special tools and no information on the reflection intensity of the radar available, we design a practical experiment of easy operation, and propose a general method for initial calibration of radar and vision points, which is a clue for the visual searching performed afterwards. Then, a novel understanding of the shadows of obstacles in the image is used in this paper for adaptive threshold selection, which is robust to illumination changes or the signs on the road surface. We aim to obtain a better comprehensive performance of the presented system through the cooperation of different parts using simplified and easily-implemented methods in terms of a system of systems.

The arrangement of this paper is as follows: in Section 2, a MMW radar and monocular vision sensor fusion approach inspired by drivers’ attention mechanisms and visual consciousness and the corresponding algorithms are described in detail. Section 3 gives the implementation of our algorithms and the experimental results. In the end, the conclusions and future plans based on current method are shown, respectively.

## 2. Proposed Fusion Approach

**Figure 1.**
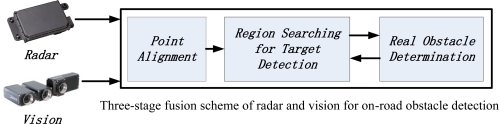
Fusion scheme of MMW radar and a monocular vision sensor.

It is known that the selective attention of human visual perception provides a referenced architecture for real-time and significant visual information processing. A prototype of a visual brain chip was designed in our lab for the development of real-time vision systems for intelligent robots based on visual attention [19]. Inspired by the computational model of visual attention [20] and drivers’ visual consciousness, the three-level fusion strategy described in Figure 1 is provided for radar-vision fusion to build a real-time system for obstacle detection, including radar-vision point alignment, region searching for potential target detection, and real obstacle determination.

The detected point targets from the MMW radar are regarded as the potential attention focuses, like the auditory or haptic stimulus for guiding human eyes’ attention. The first level is to do radar-vision point alignment. The detected target points from the radar can be thought of as clues of potential objects. Then we let the radar points be the reference centers and sequentially break up the image into small segments. This will be good for reducing or even removing the influences of complex circumstances and eventually help to speed up obstacle detection and further determine obstacles’ contour with light computational load and little processing time. For the experienced drivers’ consciousness, the boundaries of the near front or rear obstacles are the important factors for driving decision making, which is also the key for an AGV’s navigation. After the first two steps, the complex and time-consuming visual tasks can be carried out easily and fast; on the other hand, it is helpful for decreasing the false and redundant target detections from the radar. As we all know, human beings just think “Don’t hit either side of the obstacle in front” when walking, running fast or driving cars forward. Without loss of generality, the left-right edge detection of real obstacles is exemplified for testing our presented approach.

### 2.1. Radar-Vision Coordinate Calibration

**Figure 2.**
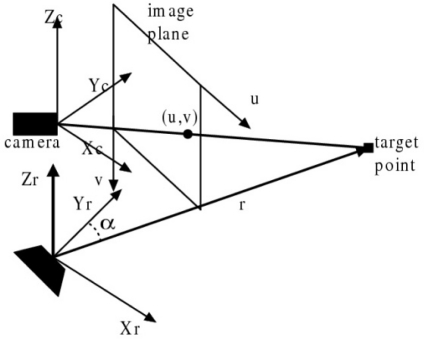
Sketch of the target point in the radar and camera coordinate systems

The target point in the coordinates of the radar and CCD camera is shown in Figure 2. (*Xr*, *Yr*, *Zr*) denotes the general MMW radar coordinate. (*Xc*, *Yc*, *Zc)* denotes the CCD camera coordinate. (*u*, *v*) is the image coordinate. The range of targets in the radar coordination is denoted as *r*, and the azimuth of the target point is represented by *α*. Generally, calibration of the CCD camera is a complex task involving many parameters. Here the radar gives out the position of a potential point target, but its exact position cannot be obtained through the CCD camera. Thus traditional coordinate transformation cannot only not solve such problems well, but also introduce additional errors into the final result.

**Figure 3.**
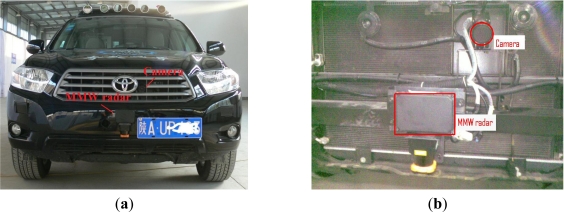
(**a**) The distribution of the MMW radar and CCD camera on our AGV platform; (**b**) the installation of radar and camera mounted on the vehicle.

Accordingly, to complete this task effectively and conveniently, this paper presents a general method for radar-vision point alignment without special tools and or the reflection intensity from radar being available. In this paper, the AGV shown in Figure 3(a) is used as the experimental platform, and the installation of MMW radar and monocular vision sensor is depicted in Figure 3(b).

The monocular camera is mounted downward so as to focus on the road surface and it will be helpful for further on-road obstacle detection algorithm design. The position of a target is represented as (*x_r_*, *y_r_*) in 2D coordinates because the radar used just gives azimuth information in 2D with no information on pitch angle in the 3D plane. The simplified calibration matrix is obtained through:

(1) $$\left(\begin{array}{c} u\\ v\\ 1 \end{array}\right)=T_{I}^{R}\left(\begin{array}{c} x_{r}\\ y_{r}\\ 1 \end{array}\right)=\left[\begin{array}{ccc} t_{11} & t_{12} & t_{13}\\ t_{21} & t_{22} & t_{23}\\ t_{31} & t_{32} & t_{33} \end{array}\right]\left(\begin{array}{c} x_{r}\\ y_{r}\\ 1 \end{array}\right)$$

where $T_{I}^{R}$ is a 3 × 3 transformation matrix, *x_γ_* = *r*sin*α* and *y_γ_* = *r*cos*α*. The above equation directly converts the radar coordinate into the image one. Six parameters of transformation matrix can be solved through the following calculation.

Let *T_i_* = [*t*_*i*1_    *t*_*i*2_    *t*_*i*3_]′, *U* = [*u*_1_    *u*_2_    ⋯    *u_n_*]′, *V* = [*v*_1_    *v*_2_    ⋯    *v_n_*]′, *I*_*n*×1_ = [1    1    ⋯    1]′ and $P=\left[\begin{array}{ccc} x_{r}^{1} & y_{r}^{1} & 1\\ \vdots & \vdots & \vdots \\ x_{r}^{n} & y_{r}^{n} & 1 \end{array}\right]$, where *n* is the number of aligned points, and ( $x_{r}^{j}$, $y_{r}^{j}$) with *j* = 1,2,…,n (n ≥ 4) is the position of the aligned point in radar coordinates. The transformation matrix $T_{I}^{R}={\left[{T^{\prime }}_{1}\;{T^{\prime }}_{2}\;{T^{\prime }}_{3}\right]}^{\prime }$ is obtained using the linear least square (LS) method as follows:

(2) $$T_{1}={\left({\mathrm{PP}}^{T}\right)}^{-1}P^{T}U$$

(3) $$T_{2}={\left({\mathrm{PP}}^{T}\right)}^{-1}P^{T}V$$

(4) $$T_{3}={\left({\mathrm{PP}}^{T}\right)}^{-1}P^{T}I_{n\times 1}$$

In this paper, the calculated transformation matrix is:

(5) $$T_{I}^{R}=\left[\begin{array}{ccc} 65.2 & -2.6 & 375.4\\ 3.3 & -2.7 & 115.9\\ 0 & 0 & 1 \end{array}\right]$$

**Figure 4.**
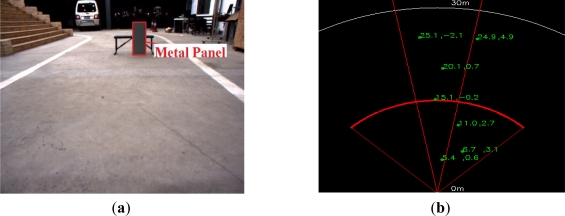
(**a**) Experimental image for radar-vision point alignment; (**b**) the corresponding radar detection map.

The commercial Delphi MMW radar has a ±45 degree Field of View (FOV) in the mid-range working mode, and ±10 degree FOV in the long-range working mode, which are indicated with the red line in a radar detection map such as Figure 4(b). It directly provides the detected point targets which are the results of original radar data clustering, and the maximum reflection intensity cannot be obtained through the Delphi ESR for the precise calibration. The exact detected target point from the MMW radar cannot easily be determined in the image, which needs special tools and maximum reflection intensities from the radar. Thus we design an experimental method of easy implementation as follows.

The calibration experiment is shown in Figure 4. An easily available square metal panel is used as the reference target as shown in Figure 4(a), whose size needs to be as small as possible but large enough to facilitate radar detection. Figure 4(b) is one of the corresponding radar detection maps projecting the real measurements in the radar coordinate, which is shown on-line using VC programming through OpenCV library, radar data translation and equipment driving programs (CAN-USB module transferring CAN data from the radar to blade server or IPC on the vehicle platform). According to the tests in a static environment, the clustered target point from the Delphi ESR reflected by the metal plane is not stationary. Generally, the clustering method employed by the radar is a nearest neighbor clustering algorithm. Based on the consideration of this feature, the centroid of the image of panel (*u_c_*, *v_c_*) was assumed as the detected target point from radar to facilitate calculation:

(6) $$u_{c}=\frac{\sum_{w}wI_{w,h}}{\sum_{w,h}I_{w,h}},v_{c}=\frac{\sum_{h}{\mathrm{hI}}_{w,h}}{\sum_{w,h}I_{w,h}}$$

where *w*, *h* are respectively the width and height of the image of the panel, and *I_w,h_* is the pixel value. The total dimension (total width and total height) of the panel image is denoted by *W* × *H*. Through the above method, radar-vision point alignment can be carried out, which will be viewed as the potential attentive points. Just because precision calibration cannot be easily obtained and the calibration results are used to give clues for the subsequent obstacle searching and verification, in some sense, the dependence on calibration errors will be weakened. Because the real position of radar point in the image cannot be precisely localized, it is relatively rough, but through our experiment, the radar point can be positioned in the image of obstacle, and this proves to be feasible and easily performed.

### 2.2. Visual Searching of Potential Obstacles

**Figure 5.**
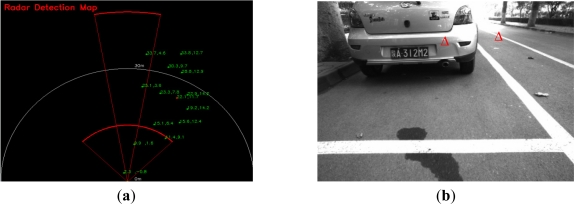
(**a**) MMW radar detection map on a campus road; (**b**) the fusion result corresponding to the near potential targets detected by radar.

After radar-vision point alignment, the targets detected by radar can be localized in the image, but this it is not very helpful for obstacle avoidance and not to mention for AGV navigation. After all, the radar can capture many targets, false or real, under urban or campus road environment conditions. The radar-vision aligned points indicate that they are potential obstacles. Figure 5(a) gives a example of the radar detection map in a campus road environment, and Figure 5(b) is the result of radar-vision alignment, which shows the potential obstacles near the host vehicle. There is a real obstacle and a false alarm. Here according to the performance of radar, we assume that all the real obstacles can be detected by MMW radar.

To exploit the clues from radar-vision alignment effectively, analysis of drivers’ visual consciousness is described as follows:

1. A driver just pays attention to road traffic within a limited area ahead of the host vehicle;
2. In the driver’s forward field of view, he is just concerned on the sizes of nearby objects, vehicles or pedestrians;
3. The objects from the bottom to top in the image mean that the relative distance between host and objects are farther, and therefore the targets detected by radar with short ranges are the main focuses;
4. As for the obstacles far away from the host vehicle, the detected target points from the radar are enough for driver’s collision alarming;
5. The long distance targets detected by the radar can hardly be projected in the image due to being hidden or too a small component of the targets appears in the image;
6. The driver’s forward sight distance is related with the driving speed. For example, the speed of vehicle is under 40 km/h, and generally the viewing distance just needs to be less than 50 m.

**Figure 6.**
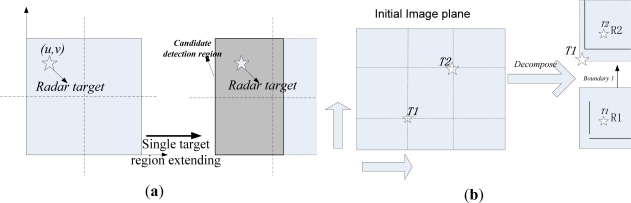
(**a**) Determination of the candidate region for single target detection; (**b**) Determination of candidate regions for two target detections.

According to the above analysis, first the detection should be limited to the road area near the host vehicle. Second, not many obstacles should be detected at one time. Then their boundaries should be determined for further precision navigation. In this paper, the road borders are assumed to be known in advance, which needs the detection of free road surface, and therefore the valid obstacle detection should be confined within this range. Therefore the radar-vision point alignments are done within this range and the visual searching is limited in this region. No more than two significant obstacles should be detected. Figure 6 describes the search strategy for potential obstacle detection in order to further decrease image processing time, especially for large image sizes.

According to our experiments, the calculated transformation matrix (5) could be used within the distance of 20 m. Figure 6(a) describes the region searching approach for single target detection. Without loss of generality, the radar-vision aligned point *(u, v)* is considered as the center of the target image. The candidate region for potential obstacle detection (*C_Region*) is obtained as follows:

(7) $$C\_\mathrm{Region}=\{\begin{array}{l} \overset{⌢}{w}\in [1,u+W/2],\overset{⌢}{h}\in [1,v+H/2]\;\mathrm{if}\;u\leq W/2,v\leq H/2\\ \overset{⌢}{w}\in [1,u+W/2],\overset{⌢}{h}\in [v-H/2,H]\;\mathrm{if}\;u\leq W/2,v>H/2\\ \overset{⌢}{w}\in [u-W/2,W],\overset{⌢}{h}\in [1,v+H/2]\;\mathrm{if}\;u>W/2,v\leq H/2\\ \overset{⌢}{w}\in [u-W/2,W],\overset{⌢}{h}\in [v-H/2,H]\;\;\;\;\;\;\mathrm{else} \end{array}$$

**Figure 7.**
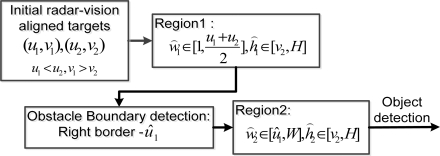
Region searching strategy for two potential targets.

Figure 6(b) describes region searching scheme in the image with two targets. The process of the searching algorithm is described in Figure 7.

### 2.3. Potential Obstacle Detection Algorithm

This paper aims to integrate a MMW radar and a monocular camera for on-road obstacle detection, which is a part of our research work on AGV navigation under urban road environment conditions. In this situation the possible obstacles will be vehicles or pedestrians. Sun [21] worked extensively on methods for on-road vehicle detection using imaging processing. In addition, Geronimo [22] summarized the pedestrian detection for advanced driver assistance systems using image processing. Budi [23] used a camera to track and identify in real-time moving persons in outdoor environments. The detection of these obstacles needs to consider different visual features so as to identify vehicles and pedestrians, respectively. An only vision-based method is time-consuming and complex. In this paper, experimental examples for obstacle detection using the fusion of radar and vision are figured out. Based on the above analysis of driver’s visual consciousness on the road, the detection should be limited to close ranges, which is helpful for reducing the number of potential obstacles and furthermore decreasing the computational loads. Then the left-right boundary of real obstacles is the driver’s visual focus, so the edges of obstacle should be determined. Researches [24–26] on road edge detection have been done well based on image processing. Thus, without loss of generality, we just assume that the road border in the image is already known for the convenience of testing. The difficult research work on free road surface detection has been done [27,28].

**Figure 8.**
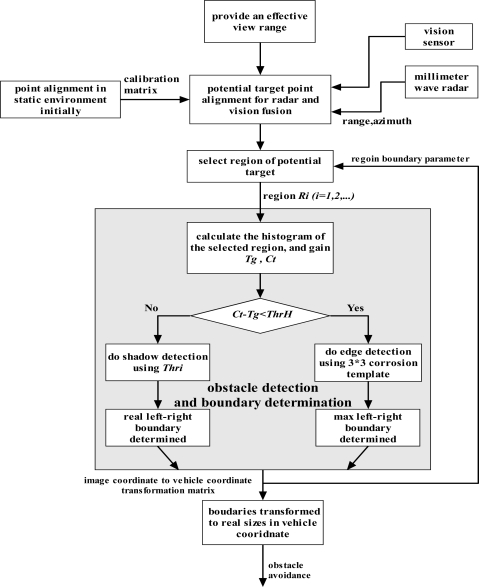
Flow diagram of obstacle detection algorithm using radar and monocular vision fusion.

**Figure 9.**
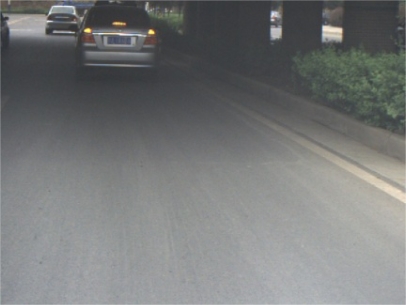
Shadow underneath the vehicle is covered by the shadow under the overhead bridge.

**Figure 10.**
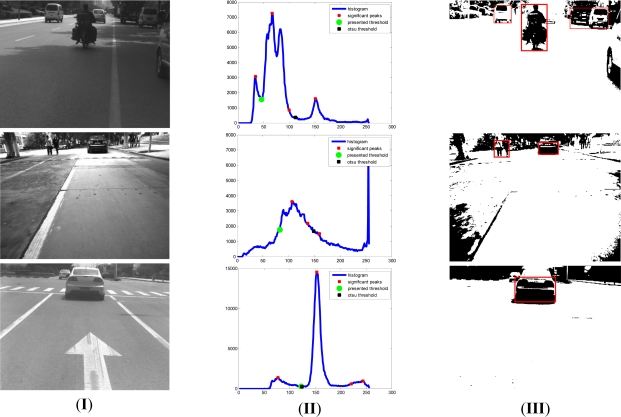
Some results of shadow detection of on-road obstacles based on our adaptive threshold selection method (**I**) Original on-road images (**II**) Results of threshold selection-the green circular point is selected by our proposed method, while the black rectangular point is gained by Otsu’s method. (**III**) Results of shadow detection of on-road obstacles based on our presented analysis of visible light imaging in the sensor.

The flow chart of our proposed algorithm for on-road obstacle detection through radar and monocular vision fusion is described in Figure 8. Shadows underneath the vehicle can be used to estimate the left-right boundary, which is a simple and continuous but probably invalid feature. The shadow exists under the cross-bridge shown in Figure 9, which could bring about difficulties for shadow detection and even make the detection fail. Here, the 492 × 656 RGB image from CCD camera is transformed into the corresponding grayscale image for the subsequent processing. As for the pedestrians, the edges of pedestrians are salient and somehow pedestrian appear as a shadow in the image shown in Figure 10. A 3 × 3 corrosion template is used for edge detection. Gray histogram *h*(*i*) is used for adaptive threshold selection, and it is obtained by Equation (8):

(9) $$h(i)=\frac{C(i)}{N}$$

where *i* = 0,1, 2, ⋯, 255 is the gray level, *N* is the total number of pixels, and *C*(*i*) is the number of the *i^th^* gray-level pixels.

Although Otsu’s method [29] is a good thresholding method for a dual-mode Gaussian model in an image, it may be ineffective in natural environments because it cannot consider the actual properties of natural images. Due to the importance of adaptive threshold selection of *Thr_i_*, this paper adopts an adaptive threshold determination algorithm based on prior research [30,31]. Firstly, the monocular camera is specially mounted so as to simplify the design of our algorithms. The downward inclined installation of monocular vision sensor on the vehicle is designed for the sake of focusing on the road surface, which could certainly make the road pixels be the main part of the image. In addition, the shadow of obstacles is given a novel understanding for the algorithm development as follows: according to the physical imaging principles of the CCD camera [32,33] and from the viewpoint of optics, it is known that objects like plants [34] may diffusely reflect, absorb and shade the light and the light intensities received by CCD sensors will be relatively lower. It will form darker parts in the corresponding locations in the gray-level image. Here the darker parts can be considered as the obstacles’ shadows, which give us the indications of potential obstacles for detection under special situations.

What is mentioned above is the core issue for radar and vision fusion proposed by us in order to detect and localize the potential targets. The radar-vision aligned point gives the clue for searching potential targets. According to our prior knowledge about the shadow underneath the vehicle, searching the shadow should be downward from the radar-vision point. When the difference between maximum value *Ct* and minimum value *Tg* of the gray histogram is less than *ThrH*, it is considered that a shadow will not be a good choice for potential obstacle detection and the edge gradient could be used easily. Here *ThrH* is set as 400. The edge detection is executed by the 3 × 3 corrosion template, and the potential target with continuous edges must be around the radar-vision aligned point. Through gradual processing of these parts, the final performance will be improved, which will decrease the demands of each part and be helpful for engineering applications.

## 3. Implementation and Results

In the experiment, some relatively simple campus/urban road environments were investigated for testing our approach and the velocity of the vehicle was at the speed of less than 30 km/h. There are few obstacles on the road and the road environment in front of the host vehicle is slow changing. Most of experimental data has one obstacle for the detection so as to verify our design. In this case, the radar data and corresponding image sequences were acquired through our AGV platform. The transformation matrix for calibration is computed using the data within the range of 20 m. Within this range, the target detected by MMW radar can be aligned well in the image, which is helpful for further vision-based obstacle detection and real obstacle verification. Thus, this paper used the radar data within a range of no more than 20 m. MATLAB code was used for programming on a windows notebook equipped with a 1.8 GHz Core Duo CPU and 2 GB RAM.

Figure 10 gives experimental examples of obstacles’ shadow detection results. In Figure 10, column I shows the original images under different road environments, column II presents the results of threshold selection for shadow detection based on our proposed approach, and shows the comparison with Otsu’s method. The black rectangle point is the threshold obtained by Otsu’s method, which will be ineffective through comparisons with the green circular point calculated through our method. Our proposed thresholding method considers the natural optical distributions shown in the image, and is more suitable for distinguishing the obstacles from the road background. In column III the potential obstacles are detected through our proposed shadow detection method. Just because our approach is based on the analysis of new analysis of shadow for on-road obstacle detection and considers intensity distributions of practical environments reflected in the vision sensor, our approach is more reasonable and adaptive under different environments. From the results, we can find that it is robust to changing illumination and thick road signs on the road and it can be helpful for the determination of obstacles’ boundaries after the radar-vision fusion.

**Figure 11.**
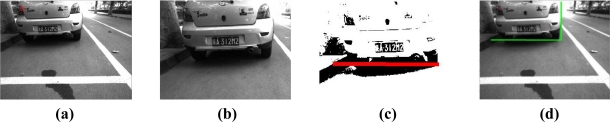
(**a**) Original experimental image; (**b**) Candidate searching region determination; (**c**) Shadow detection result; (**d**) Right border determination.

**Figure 12.**
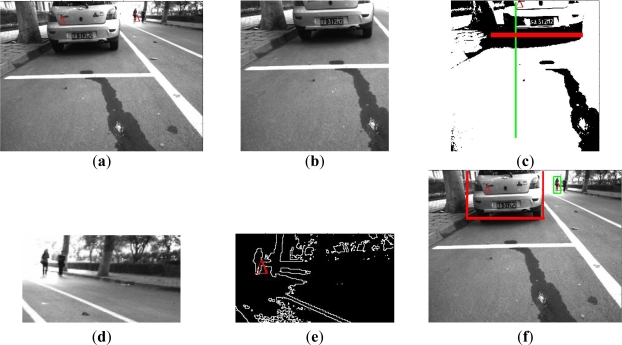
Detection of obstacle boundaries: (**a**) radar-vision point alignment (**b**) one candidate region (*R1*) with an obstacle (**c**) shadow detection result in*R1* (**d**) another candidate region (*R2*) with an obstacle (**e**) edge detection result in *R2* (**f**) final results of boundary determination.

Figure 11 shows the detection process of a single potential obstacle on the basis of our proposed radar-vision fusion method. Figure 11(a) gives the radar-vision point fusion result in the original image, and Figure 11(b) gives the result of region searching in the original image. Figure 11(c) shows the detection result in the segmented region, and then it is mapped in the original image shown as Figure 11(d). Figure 12 gives an example of obstacle boundary determination based on the proposed algorithm above, and describes the detection processing of two potential obstacles according to the presented method above. Figure 12(a) is the result of radar-vision point alignment. Figure 12(b,d) is two candidate regions for potential obstacle detection, respectively, through our visual searching strategy. Figure 12(c,e) is the shadow detection result and edge determination, correspondingly. Then we map the above detection results in the original imaging and the boundaries of obstacles are determined in Figure 12(f).

**Figure 13.**
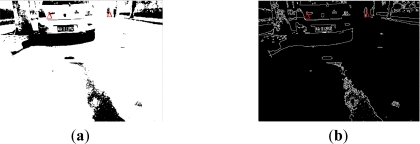
(**a**) Shadow detection result without segmentation of potential region (**b**) Edge detection result without segmentation.

The results of shadow and edge detection using the original image are shown in Figure 13. We can find that the edge information of the vehicle is not helpful for the obstacle detection or even its boundary determination. Additionally, comparing with Figure 12, it is not difficult for us to understand that through visual searching the candidate region is divided into two parts for the sake of decreasing the influence of another obstacle in the image. We can believe that it is definitely valuable for the detection in the higher resolution image. About 1,000 frames of experimental images were used for our test. The averaged processing time is less than 100 ms, which will easily satisfy the real-time demand.

## 4. Conclusions and Future Plans

Because MMW radar can be used in all-weather applications and its performance cannot be degraded by dusts and fogs, integration of MMW radar with vision sensors has been attracting more and more attention, not only for AGVs but also for planetary rovers or military applications. This paper is an extensive work based on our prior conference presentation [35], and we have developed the algorithms in detail and done more experimental work. We aim to provide a feasible solution in terms of a system of systems so as to obtain the better comprehensive performance of the final system through combinations of simplified methods and implement real time on-road applications for AGVs in the future. This paper provides a novel experimental scheme integrating MMW radar with a monocular vision sensor for on-road obstacle avoidance applications, which is a part of our research work on AGV navigation. With no original information of reflection intensity from radar and special tools unavailable, an easily-implemented experiment and calculations for MMW radar and vision point alignment are presented. Visual target searching strategy with the aid of radar-vision aligned points is proposed. From the viewpoint of photoelectric imaging, a novel understanding of shadows of obstacles in the image is utilized for the detection. The exemplified obstacle detection approach is designed for the determination of obstacle boundaries. Experimental data under simple well-paved campus/urban environment conditions were used frame by frame to test the fusion method mentioned above. The results show that the presented approach is simple but feasible and valid for on-road obstacle detection from an engineering point of view, but in a crowded road environment it is difficult for the discrimination by radar and vision, and the algorithm will fail. According to our prior work on attention-based visual brain chip design [19], our method is potentially suitable for hardware implementation.

However, due to the complexity and intensive dynamics of on-road environments, the method should be further improved through addition of more useful features for reliable on-road obstacle detection so as to achieve collision avoidance and precise navigation. The calibration errors in our method should also receive more quantitative considerations. The threshold selection approach used in this paper has to be given more considerations so as to make it effective in common environments.
